# Brain-Region Specific Metabolic Abnormalities in Parkinson’s Disease and Levodopa-Induced Dyskinesia

**DOI:** 10.3389/fnagi.2020.00075

**Published:** 2020-03-17

**Authors:** Changwei Yang, Tingting Zhang, Wuqiong Wang, Yilan Xiang, Qun Huang, Chenglong Xie, Liangcai Zhao, Hong Zheng, Yunjun Yang, Hongchang Gao

**Affiliations:** ^1^School of Pharmaceutical Science, Wenzhou Medical University, Wenzhou, China; ^2^Department of Radiology, The First Affiliated Hospital of Wenzhou Medical University, Wenzhou, China; ^3^Department of Neurology, The First Affiliated Hospital of Wenzhou Medical University, Wenzhou, China

**Keywords:** Parkinson’s disease, levodopa-induced dyskinesia, metabolism, neurotransmitter, Glu-Gln-GABA cycle

## Abstract

Several lines of evidence point to alteration in brain metabolic homeostasis in Parkinson’s disease (PD) and levodopa-induced dyskinesia (LID), yet the metabolic mechanism in different brain regions underlying PD and LID remains largely unknown. The present study aimed to uncover the metabolic pathways across anatomical regions in the brain of PD and LID. Using an NMR-based metabolomic approach, we generated the metabolomics profiling data from six different brain regions of PD rats and following the onset of LIDs. The diversity of metabolite patterns across the brain and its relation to PD and LID were further investigated through principal component analysis (PCA) and multivariate general linear model. Compared with control rats, dopamine loss in PD rats produced a marked and persistent metabolic disturbance in neurotransmitter metabolism and energy pathway, resulting in a metabolic imbalance among different brain regions. In LID rats, levodopa replacement did not restore the midbrain-striatum metabolic crosstalk and metabolic disturbance throughout the brain was involved in levodopa related involuntary movements. Most notably, the midbrain and right cortex were identified as the primary regions of metabolic abnormalities in PD and LID rats. Neurochemical differences in metabolic phenotypes were mainly defined by various neurotransmitters including glutamate, glutamine and aspartate. Accordingly, we found that the PD and LID rats exhibited lower levels of synaptophysin (SYP), a marker for synaptic plasticity, compared with control rats. These findings provide key insights into the metabolic mechanism underlying PD and LID by defining brain-region specific metabolic phenotype, with implications for developing targeted therapies.

## Introduction

Parkinson’s disease (PD) is a common age-related neurodegenerative disorder due to the loss of dopaminergic neurons in the substantia nigra par compacta (SNpc) thereby leading to the dopamine depletion in the striatum (Huang et al., 2019). The clinical symptoms of PD are mainly classified into motor abnormalities characterized by static tremor, rigidity, bradykinesia, postural instability and non-motor symptoms, such as cognitive impairment and gut dysfunction (Lee et al., 2019). So far, L-3,4-dihydroxyphenylalanine (L-dopa) remains to be the most effective drug for symptomatic treatment of Parkinson (Smith et al., 2014). However, the adverse effects of long-term L-dopa treatment vary, such as levodopa-induced dyskinesia (LID), which appears in approximately 40% of PD patients after 5 years L-dopa therapy and up to 90% within 10 years (Pourmirbabaei et al., 2019; Sellnow et al., 2019). Furthermore, the uncontrollable LID has been reported to dramatically affect the quality of patients’ life and greatly augment the cost of health care for which no satisfying treatment is available (Dodel et al., 2001; Chapuis et al., 2005; Hechtner et al., 2014). In light of this urgent medical need for rapidly aging populations, there is an urgent need to identify the mechanism underlying PD and LID.

Metabolic abnormality has long been considered to be involved in a lot of diseases, such as neurodegenerative diseases including PD (Shao and Le, 2019) and Alzheimer’ disease (Van Bulck et al., 2019), diabetes mellitus (Del Coco et al., 2019), chronic obstructive pulmonary disease (Lamonaca et al., 2017). ^1^H-NMR based metabolomics has proven to be a key for the characterization of metabolic profiles relevant to brain function and disease (Ivanisevic and Siuzdak, 2015). Our previous studies demonstrated that metabolic abnormalities in the brain participated in DM-associated cognitive dysfunction (Zheng et al., 2017) and pathogenesis of Alzheimer’s disease (Zheng et al., 2018b). And increasing evidence in recent researches has supported the idea that metabolic abnormality may be causal to PD and LID, though it is unclear how metabolic disruption affects striatal activity *in vivo*. In our previous study, ^1^H NMR based metabolomics was applied to evaluate the metabolic signatures and pathways involved in the onset and progression of PD, which indicate that neuron loss and motor function impairment in induction PD mice may be linked to overactive glutamate-glutamine cycle and altered membrane metabolism in the striatum (Lu et al., 2018). There is, however, a dearth of information on understanding the metabolic and cellular mechanisms underlying motor impairment in LID.

Historically, studies of PD and LID have been focused on the striatum, since it is acknowledged that the dopamine depletion in the striatum result from degeneration of dopaminergic neurons is the main cause of PD (Hornykiewicz, 2006; Huang et al., 2019), and the prevailing hypothesis is that subsequent replacement with levodopa causes excessive direct pathway activity in the striatum. For example, Cenci et al. (1998) analyzed the correlations between the dyskinetic symptoms of LID and neurotransmitter-related mRNA expression in the basal ganglia and found that striatal overexpression of prodynorphin- and glutamic acid decarboxylase participated in the occurrence of LID. Santini et al. (2007) found hyperactivation in the striatal medium spiny neuron of cAMP/PKA/DARPP-32 and ERK signaling played a critical role in L-dopa induced dyskinesia. Crucially, L-dopa replacement has little effects on axial symptoms of PD, indicating that disruptions in another neurotransmitter metabolism other than dopamine, even other brain regions beyond the striatum could mediate these PD and levodopa-evoked behaviors. Again, some indirect evidence supports this hypothesis: once LID develops, a given dose of levodopa relieves parkinsonism but produces dyskinesia.

If other brain regions were indeed involved in the onset and progression of PD and LID, then targeted therapies could be more effective than levodopa alone. Therefore, the creation of a detailed map of the human neural metabolic pathway across different brain regions will help to build a knowledge base to unravel complex brain functions. In the present study, we took advantage of a ^1^H NMR-based metabolomics approach to characterize the involvement of region-specific metabolic phenotype in the brain of PD and following LID rats.

## Materials and Methods

### Animals

Male Sprague–Dawley rats (180–220 g) purchased from the SLAC Laboratory Animal Company Limited (Shanghai, China) were housed under the standard laboratory conditions (controlled temperature/humidity condition, a normal 12/12-h light/dark schedule with the lights on at 08:00 a.m.), in the Laboratory Animal Center of the First Affiliated Hospital of Wenzhou Medical University (Wenzhou, China). All rats were given free access to standard chow and water during the whole experimental process. All experimental operations were performed strictly in accordance with the National Institutes of Health Guide for the Care and Use of Laboratory Animals and were approved by the Institutional Animal Care and Use Committee of Wenzhou Medical College (wydw2018-015).

### 6-Hydroxydopamine (6-OHDA) Rat Model of PD and Levodopa-Induced Dyskinesia

Animals were weighed and randomly divided into control (*n* = 8) and lesion groups (*n* = 20). All rats in lesion group stereotaxically received 4 μl of 6-OHDA (4 μg/ μl, dissolved in 0.9% physiological saline involving 0.2% ascorbic acid) injection at each coordinate in the right medial forebrain bundle (MFB) and the rats in control group received sham-operation with physiological saline at the same locations. The coordinates calculated by the rat brain atlas were as previously reported (Xie et al., 2014): AP −4.4 mm, ML −1.2 mm, DV −7.8 mm; AP −3.7 mm, ML −1.7 mm, DV −7.8 mm. The tooth bar was set to −2.4 mm. After 3 weeks of lesioning, rats in the lesion group received intraperitoneal injection of apomorphine to induce contralateral rotations, and rats with at least seven full contralateral turns/min were selected as successful PD rats (Yang et al., 2012). The successful PD rats were then randomly divided into PD group (*n* = 10) and LID group (*n* = 10). In the next 3 weeks, rats in LID group received a daily intraperitoneal injection of L-dopa (15 mg/kg) together with benserazide (3.75 mg/kg) for 3 weeks (Zhang et al., 2014) and the rats in Con and PD group were given equal vehicle during the same period. The body weights of rats were constantly monitored during the process of the study.

### Behavioral Test

The onset and severity of LID were evaluated according to a highly validated abnormal involuntary movements (AIMs) scale on days 1, 4, 7, 10, 14, 17 and 20 of L-dopa/benserazide treatment (Lundblad et al., 2002; Mellone et al., 2019). Briefly, AIMs were conducted from 20 min to 120 min after L-dopa or vehicle administration and each rat was continuously observed for 1 min every 20 min each time. The AIMs scores were recorded from 0 to 4 according to the incidence of three subtypes of AIMs (axial, limb and orolingual): 0 = absent; 1 = present less than or equal to 30 s; 2 = present more than 30 s; 3 = present continuously but suppressible by external stimulus; 4 = present continuously but not suppressible by external stimulus.

The forelimb function test is a widely used behavior test to assess the forelimb motor function of rats. The test was performed at days 3, 8, 13, 18 of L-dopa/benserazide treatment and all the process was carried out in a glass beaker (diameter = 22 cm, height = 35 cm) to record the number of wall contacts of right/left forelimb of rats. 1 h after L-dopa or vehicle administration, the forelimb function test was evaluated 5 min every 20 min for four times. The final value was shown as the percentage wall contacts of the contralateral forelimb of 6-OHDA lesion (left forelimb) compared with total wall contacts of bilateral forelimbs (Schallert et al., 2000).

### Immunofluorescence Staining

Three rats of each group were processed for immunofluorescence staining studies. In brief, rats were anesthetized with 10% chloral hydrate (0.35 ml/100 g) first after the last L-dopa administration and then perfused transcardially with normal saline followed by a fixing solution containing 4% paraformaldehyde for 30 min. The brains were carefully removed, fixed in 10% buffered formalin overnight, and embedded in paraffin. The brain tissues were cut into coronal sections with a thickness of 5 μm. The paraffin sections were incubated with Anti-Synaptophysin (SYP) antibody (Abcam, 1:200) overnight at 4°C and rinsed in PBS followed by incubation with Alexa Flour™ 488 goat anti-rabbit lgG (H + L; Invitrogen, 1:500). Subsequently, sections were rinsed again in PBS, stained with DAPI (Southern Biotech) and observed using a fluorescence microscope (Nikon, Tokyo, Japan).

### Sample Collection and Metabolite Extraction

The remaining rats were sacrificed by decapitation after a 3-weeks L-dopa administration, with the whole brain tissue dissected into midbrain, cortex, striatum, hippocampus, cerebellum, and hypothalamus immediately. All the brain tissues were put into liquid nitrogen at once and then stored at −80°C until use. The metabolite extraction was referred to as our previous method (Liu et al., 2018). Briefly, the frozen tissues were weighted into an Eppendorf tube, Following the homogenization by electric homogenizer with ice-cold methanol (4.0 ml/g) and ultrapure water (0.85 ml/g). The mixture was homogenized again with 2 ml/g of chloroform, then again with 2 ml/g of chloroform and 2 ml/g of ultrapure water using a vortex mixer, placed on ice for 15 min, and centrifuged at 10,000 *g* for 15 min at 4°C. Finally, the supernatant was carefully transferred into a new Eppendorf tube, lyophilized for 24 h, and stored at −80°C until NMR analysis.

### NMR Spectroscopy

The lyophilized extract was dissolved into 0.6 ml of 99.5% D_2_O containing 0.05% of sodium trimethylsilyl propionate-d4 (TSP), in which D_2_O provided a field-frequency lock, and TSP was used as the chemical shift reference. All ^1^H NMR spectra of brain tissue were acquired on the Bruker Avance III 600 MHz NMR spectrometer with a 5-mm TXI probe (Bruker BioSpin, Rheinstetten, Germany) at 298 K. The one-dimensional ^1^H NMR spectra of right striatum and SN were acquired using a standard single-pulse sequence with water signal presaturation (ZGPR) with number of scans (256), acquisition time (2.65 s/scan), date points (64 k), spectral width (12,000 Hz) and relaxation delay (6 s). Then, the ^1^H NMR spectra were referenced to the TSP peak at 0.0 ppm and manually adjusted phase and baseline using Topspin (v2.1 pl4, Bruker Biospin, Germany).

### Data Processing of NMR Spectra and Multivariate Pattern Recognition

NMR spectra were reduced to integrated regions with a width of 0.01 ppm (bin) corresponding to the region of δ 10–0.5. The regions of 4.7–5.2 ppm (water) was removed to eliminate artifacts related to the residual water resonance. The remaining spectral segments were then normalized to the total sum of the spectral intensity to partially compensate for differences in the concentration of many metabolites in the samples. Before multivariate data analysis, the integral values were mean-centered and Pareto-scaled (Shao et al., 2014). NMR data sets were imported into SIMCA-P + 12.0 software (Umetrics, Umea, Sweden) for multivariate statistical analysis, including principal component analysis discriminant analysis (PCA-DA) and partial least squares-discriminant analysis (PLS-DA). And another data reduced to integrated regions with a width of 0.0015 ppm width corresponding to the region of δ 10–0.5 were used for quantitative analysis.

### Real-Time RT-PCR

The Real-time RT-PCR procedure and data analyses were carried out as previously described (Yang et al., 2019). The total RNA was extracted from the right cortexes using standard TRlzol reagent (Invitrogen, Carlsbad, CA, USA) and converted into cDNA following the instruction of PrimeScript™ RT reagent Kit (Takara, RR037A). Individual samples were measured in triplicate. Real-time-PCR was performed on an ABI 7500 Fast Real-Time PCR system using TB Green™ Premix Ex Taq™ II (RR820A). Primer sequences as follows: Actin (5′-ACCCGCGAGTACAACCTTC-3′/5′-ATGCCGTGTTCAATGGGGTA-3′), SYP (5′-GCTAAAAGCAGGAGGGCGTA-3′/5′-GCACAGGAAAGTAGGGGGTC-3′). Relative expression levels were measured with the 2^−ΔΔCt^ method.

### Statistical Analysis

Independent sample *t*-test or one-way ANOVA was applied to compare the difference among different groups. Statistical analyses were performed using SPSS (version 25, IBM, USA). Data were exhibited as mean ± SEM and *P*-values less than 0.05 were considered statistically significant.

## Results

### Behavior Tests in Experimental Models of PD and LID

**Figure 1:**
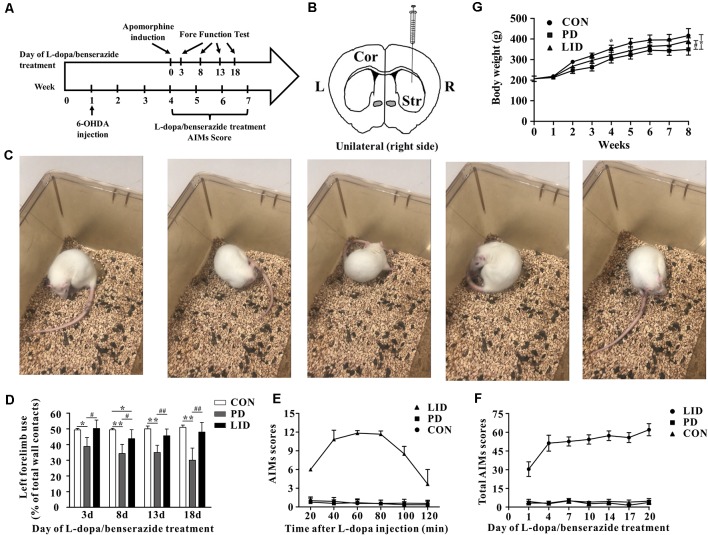
Behavior performance of the experimental models of Parkinson’s disease (PD) and levodopa-induced dyskinesia (LID). **(A)** The time scale of the experiments: 6-OHDA lesioning, L-dopa/benserazide treatment, and behavioral tests. **(B)** Schematic of stereotactic injection of 6-hydroxydopamine (6-OHDA), the rats in the control group received sham-operation with physiological saline at the same location (*n* = 8–10 for each group). **(C)** Apomorphine-induced rotation test. **(D)** Forelimb function tests at days 3, 8, 13, 18 of L-dopa/benserazide treatment. **(E)** AIMs 120-min time-score plot of three groups after L-dopa injection. **(F)** Total AIMs scores plot of three groups at Days 1, 4, 7, 10, 14, 17 and 20 of L-dopa/benserazide treatment. **(G)** Body weight over time of rats from three groups. *Means the difference between Con group and experimental groups (*t*-test); ^#^means the difference between PD and LID group (*t*-test). *P < 0.05, **P < 0.01; ^#^*P* < 0.05, ^##^*P* < 0.01.

To mimic dyskinesia of PD patients, male SD rats were unilaterally injected with 6-OHDA in the right MFB, causing a nearly complete depletion of ipsilateral dopamine, while the rats in Con group were got same Sham-operation but injected with vehicle (Figures 1A,B). Three weeks later, motor symptoms were validated using apomorphine-induced rotation test (Figure 1C). Then the LID rats were intraperitoneally administrated L-dopa (15 mg/kg, i.p.) with benserazide (3.75 mg/kg, i.p.) once a day for 3 weeks and observed progressive AIMs. Forelimb function test was conducted at days 3, 8, 13, 18 after L-dopa/benserazide treatment, results showed that the percent of left forelimb use of total wall contacts of rats in Con group is around 50%, while the percent of left forelimb use was significantly reduced in PD rats with a partial lesion of the nigrostriatal pathway. After the treatment of L-dopa, the percent of left forelimb use of rats in the LID group was significantly increased compared to that in the PD group (*P* < 0.05, Figure 1D). These results suggested that PD rats showed reduced forelimb motor function, and L-dopa treatment is quite effective in alleviating PD behavioral symptoms.

However, we found that L-dopa treatment caused both dyskinesia and contralesional rotations. Abnormal involuntary movements (AIMs) score, a standard behavior performance score system for dyskinesia, was significantly higher in the 6-OHDA-lesioned rats after 3 weeks of L-dopa administration (Figure 1E). We observed that AIMs immediately appeared at 20 min after L-dopa injection and did not attenuate until 80 min and then faded away after 120 min. Besides, we continuously monitor the total AIMs scores at days 1, 4, 7, 10, 14, 17 and 20 of L-dopa/benserazide treatment (Figure 1F).The results showed that total AIMs scores of LID rats gradually increased with time went by, while that of rats in Con and PD group had no obvious changes.

In addition, we also questioned whether the growth of rats was affected by the disease. A repeated-measures ANOVA revealed a significant time and treatment interaction effect in the body weight (*P* = 0.01). To be specific, the bodyweight of PD rats was significantly reduced after 6-OHDA administration, compared with the control group. After L-dopa administration for 3 weeks, the bodyweight of LID rats increased significantly compared with PD rats (*P* < 0.05, Figure 1G), while no significant difference was found between Con and LID rats.

### NMR-Based Metabolic Profiling of Different Brain Regions

**Figure 2:**
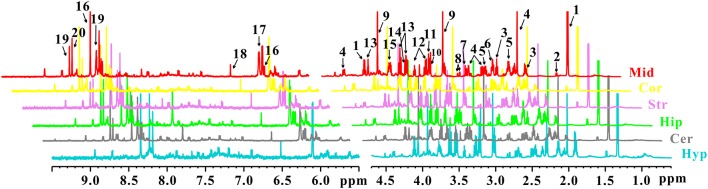
^1^H NMR spectra of different brain sections obtained from control, PD and LID rats: Mid, midbrain; Cor, cortex; Str, striatum; Hip, hippocampus; Cer, cerebellum and Hyp, hypothalamus. 1. Lactate (Lac); 2. Alanine (Ala); 3. GABA; 4. N-acetyl aspartate (NAA); 5. Glutamine (Gln); 6. Glutamate (Glu); 7. Dimethylamine; 8. Aspartate (Asp); 9. Creatine (Cre); 10. Choline (Cho); 11. Glycerol-phosphocholine (GPC); 12. Taurine (Tau); 13. Myo-inositol (Myo); 14. Glycine (Gly); 15. Glu/Gln; 16. Inosine (Ino); 17. ADP/AMP; 18. Fumarate (Fum); 19. AMP; 20. IMP.

In order to characterize the dynamic changes of metabolites throughout the brain, NMR-based metabolic profiling of different brain regions was performed among control, PD and LID rats. Typical ^1^H-NMR CPMG (600 MHz) spectra of different brain regions (midbrain, cortex, striatum, hippocampus, cerebellum, and hypothalamus, respectively), obtained from control, PD and LID rats are presented in Figure 2. Assignments were performed according to our previous studies (Zheng et al., 2017, 2018a) using the Chenomx NMR suite 7.0 (Chenomx Inc., Edmonton, AB, Canada). A total of 20 metabolites were identified, which can be detected in all six brain regions (Figure 2). Although the NMR spectra appeared similar among different brain regions, there were striking differences in peak intensities for different brain regions. The metabolites identified included lactate, alanine, GABA, NAA, glutamine, glutamate, dimethylamine, aspartate, creatine, choline, glycerol-phosphocholine, taurine, Myo-inositol, glycine, Glu/Gln, Inosine, ADP/AMP, fumarate, AMP and IMP.

### Multivariate Analysis of NMR Data

**Figure 3:**
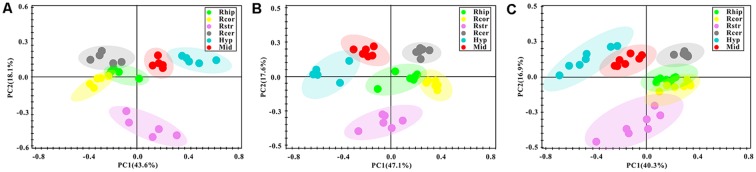
Overview of metabolic changes in six different brain regions of Con **(A)**, PD **(B)** and LID **(C)** rats.

After the pre-processing of the NMR spectra, including the bucketing process and total sum normalization to minimized small differences due to sample concentration among samples, NMR data was subjected for multivariate statistical analysis including unsupervised (PCA) and supervised (PLS-DA) multivariate statistical methods. As a first attempt, the PCA was used to quantitatively compare the global metabolomics profiling data collected from the six different brain regions of control (Figure 3A), PD (Figure 3B) and LID (Figure 3C) rats. The PCA score plot obtained for control rats revealed good separation among the different brain regions, especially along the first principal component PC1 (with PC1 and PC2 accounted for 43.6% and 18.1% of the total variance, respectively, Figure 3A). PCA score plot obtained for PD and LID rats showed similar, distinct separation among different brain regions (Figures 3B,C). As showed in Figure 3, the metabolic pattern in the striatum appeared as separate significantly from other brain regions, especially along the second principal component PC2. Whereas the midbrain and right cortex distribution was significantly different both in PD and LID rats compared with control rats, indicating midbrain-cortex crosstalk was involved in the behavioral symptoms of PD and LID.

### Brain Regional Specific Metabolic Disturbance Was Involved in Parkinson’s Disease

**Figure 4:**
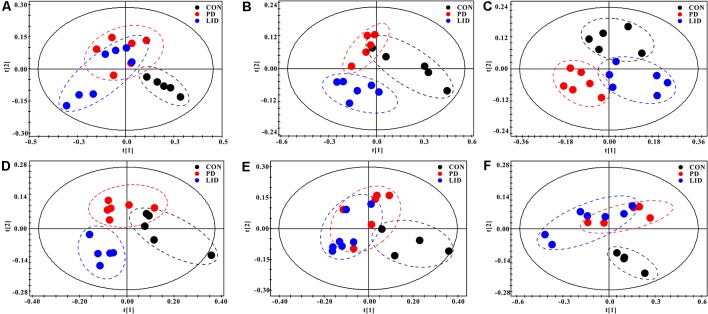
PLS-DA score plots of Con, PD and LID groups in the different brain regions:** (A)** Mid, midbrain; **(B)** Rstr, right striatum; **(C)** Rcor, right cortex; **(D)** Rhip, right hippocampus; **(E)** Rcer, right cerebellum; **(F)** Hyp, hypothalamus.

**Figure 5:**
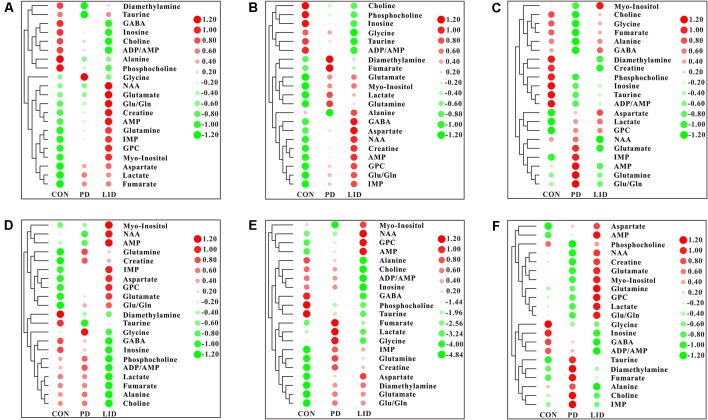
Heatmap displaying the identified metabolites in six different brain regions obtained from the brain of Con, PD and LID rats: **(A)** Mid, midbrain; **(B)** Rstr, right striatum; **(C)** Rcor, right cortex; **(D)** Rhip, right hippocampus; **(E)** Rcer, right cerebellum; **(F)** Hyp, hypothalamus.

To further explore discriminating features and potential biomarkers in brain function and disease, the supervised PLS-DA analyses were applied for six different brain regions from three groups (Figure 4). Moreover, a mixed-model analysis was applied to evaluate the effects of dopamine loss and L-dopa replacement on metabolite alterations in the PD and LID rats and the detailed results were shown in Figure 5 and listed in Supplementary Tables S1–S6. As predicted, a clear separation between control and PD rats was observed in the midbrain (Figure 4A) and striatum (Figure 4B). Accordingly, using hierarchical clustering on the profile of the identified 21 metabolites, the metabolic profile of midbrain and striatum was strictly separated between control and PD rats, several interesting metabolite clusters became apparent as well (Figures 5A,B). One of these clusters contained glutamate and glutamine, and this alteration in the glutamate metabolism pathway reflected neurotransmitter homeostasis in the brain (Bak et al., 2006). In addition, various products of energy metabolism pathways (e.g., taurine, lactate, and fumarate) in the striatum were altered in PD rats (Figure 5B).

Surprisingly, the metabolic profile of PD rats was also separated significantly in the other four brain regions: right cortex (Figure 4C), hippocampus (Figure 4D), cerebellum (Figure 4E) and hypothalamus (Figure 4F), respectively. The potential biomarkers, which are responsible for the separation were observed in the corresponding heatmap, respectively (Figures 5C–F). In summary, nine assigned metabolites with a significant difference were identified between Con and PD rats (*P* < 0.05), including lactate, glutamine, GABA, aspartate, glycerol-phosphocholine, fumarate, Glu/Gln, glycine and taurine. Of note, the midbrain and cortex were the main brain regions that underwent significant metabolic disruption between the Con group and PD groups. These observations suggest that dopamine loss in PD rats produces a marked and persistent metabolic disturbance in neurotransmitter metabolism and energy pathway, resulting in a metabolic imbalance among different brain regions.

### L-Dopa Induces Brain Regional Specific Metabolic Disturbance During LID

The standard model predicts that by increasing striatal dopamine, L-dopa is hypothesized to restore the normal balance of striatal activity. To test the metabolic activity of the whole brain during LID, PLS-DA analyses were also performed for control and LID rats (Figure 4). PLS-DA score plots showed an even better separation in all the six brain regions than that observed comparing control and PD rats, especially in the midbrain (Figure 4A), striatum (Figure 4B), and right cortex (Figure 4C). Quantitative analysis on the metabolites from control and LID groups with *T*-test, total 10 significantly changed metabolites were detected between Con and LID rats, namely lactate, phosphocholine, aspartate, glycerol-phosphocholine, GABA, fumarate, glutamine, Myo-inositol, AMP, and alanine (Figure 5, Supplementary Tables S1–S6). Counter to our expectation, we found that the striatal metabolism was not restored in response to L-dopa, furthermore, during LID, relatively higher lactate, aspartate, glutamine, AMP and Myo-inositol was observed in midbrain and striatum, compared with that of the control group (Figures 5A–C). These findings confirmed that L-dopa replacement did not restore the midbrain-striatum metabolic crosstalk and metabolic disturbance throughout the brain was involved in levodopa related involuntary movements.

### Glutamate-Glutamine Metabolic Cycle in Parkinson’s Disease and LID

**Figure 6:**
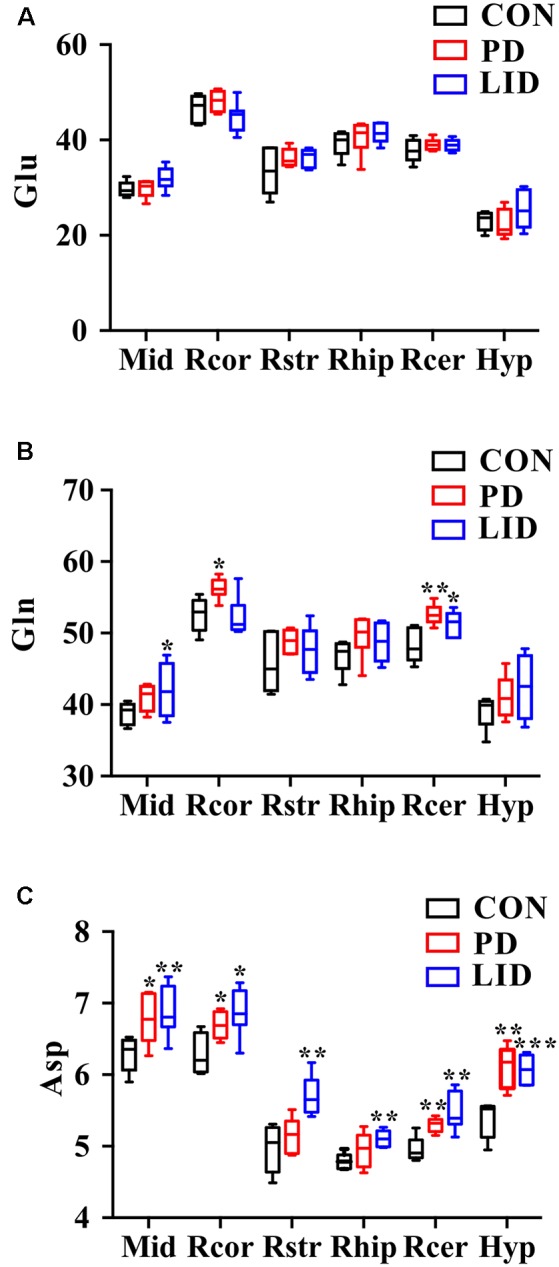
Changes in neurotransmitter metabolism-related metabolites in different brain regions. **(A)** Glu, glutamate; **(B)** Gln, glutamine; **(C)** Asp, aspartate. Differences between Con and experimental groups were analyzed using univariate ANOVA, *P < 0.05, **P < 0.01, ***P < 0.001.

Since these results point toward neurotransmitter dysregulation as an underlying feature both in PD and LID, we sought to assess how the glutamate-glutamine cycle was affected by the disease in different brain regions (Figure 6). Remarkably, we found that the concentration of glutamate in the PD and LID groups was higher than that in the Con group, except for the hypothalamus in the PD group and the right cortex in the LID group (Figure 6A). Besides, the PD rats had a higher glutamine concentration than Con rats in all six brain regions, especially in the right cortex and right cerebellum (Figure 6B). The concentration of glutamine varied in the LID group compared to the Con group, which was significantly increased in the midbrain and right cerebellum (*P* < 0.05). More interestingly, compared with the Con rats, the PD rats had a significantly higher concentration of aspartate in the midbrain, right cortex, right cerebellum and right hypothalamus, and a significantly increased of aspartate concentration occurred in all brain regions of the LID rats (Figure 6C). These results provide further evidence that glutamate-glutamine metabolism is dysregulated in D and LID.

### Synaptic Plasticity Is Impaired in Parkinson’s Disease and LID

**Figure 7:**
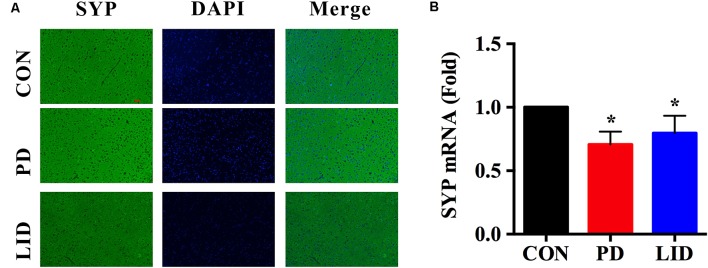
Gene and protein expression of synaptophysin (SYP) in the right cortex of control, PD and LID rats. **(A)** Immunofluorescence staining of SYP in the cortex (*n* = 3–4 for each group): labeling for SYP (Green) and staining nuclei with DAPI (blue). Magnification: X400. Scale bar: 100 μm. **(B)** Real-time RT-PCR results showed that the expression of SYP in the right cortex in the experimental groups were significantly decreased compared with that in the control group. Differences among the three groups were analyzed using univariate ANOVA. *P < 0.05 is compared with control group.

It was noticed that there was a disorder of neurotransmitters in the PD and LID rats, we wondered if the synaptic plasticity, which controls the release and recycle of neurotransmitters was also involved in the PD and LID disease. SYP, a standard presynaptic marker, which was responsible for synapse formation and neurotransmitter recycle (Wu et al., 2015). Immunofluorescence staining was performed to visually evaluate the expression of SYP in the right cortex of PD and LID rats. The immunofluorescence staining results showed that the density of SYP in the right cortex of PD and LID rats was lower than that in the Con rats (Figure 7A). Accordingly, the results of real-time RT-PCR also showed that the gene expression of SYP in the right cortex of PD and LID rats was significantly decreased compared to Con rats (Figure 7B).

## Discussion

The potential metabolic mechanisms of PD and LID are not largely elucidated and keep a great challenge for the therapy of PD (Wang et al., 2014). In the present study, the ^1^H NMR-based metabolomics approach was performed to present the first widespread investigation of metabolic profile throughout the brain in a 6-OHDA-unilaterally-lesioned rat model. We found that: (1) dopamine loss in PD rats produces a marked and persistent metabolic disturbance in neurotransmitter metabolism and energy pathway, resulting in a metabolic imbalance among different brain regions; (2) L-dopa replacement did not restore the midbrain-striatum metabolic crosstalk and metabolic disturbance throughout the brain was involved in levodopa related involuntary movements; and (3) further multivariate general linear model identified that a significant perturbation of Glu-Gln-GABA cycle in midbrain and right cortex of both PD and LID rats, accompanied by reduced synaptic plasticity.

Although many investigators have posited that energy metabolism is critical to maintaining the normal brain function (Bélanger et al., 2011), previous studies of the metabolic mechanism of PD and LID had largely focused on the midbrain and striatum, while the global metabolic profile in the adult brain has not been extensively described. Consistent with a midbrain-striatum mediated mechanism of behavior modulation, the pattern of energy metabolism in midbrain and striatum we identified in the current study is in-line with what described in numerous studies of both human and rodent models (Zheng et al., 2016, 2017). Notably, we found a significantly increased lactate level in the right striatum of 6-OHDA-lesioned PD and LID rats’ respect to Con rats, which confirms previous studies that identified lactate served as a spare energy substrate and was critical in the brain function (Proia et al., 2016). Besides, the concentration of alanine of LID rats was significantly decreased compared with Con rats. Interestingly, compared with Con rats, the fumarate level was significantly decreased in the right cortex of PD rats and in the right hippocampus of LID rats. Taken together, these results suggest that while regional specific metabolic disturbance is a feature of PD, L-dopa replacement did not restore the midbrain-striatum metabolic crosstalk may still contribute to levodopa-evoked facilitation of movement in LID.

The multivariate analysis performed on brain metabolic profile also identified a significant difference in the levels of glutamate (Glu), glutamine (Gln) and aspartate (Asp) in PD and LID rats with respect to control rats. Glu is the main excitatory neurotransmitters which can enhance neuronal excitability whereas GABA is the primary inhibitory in the central nervous system (Rage et al., 1992). Glutamate is the metabolic precursor of GABA, which can be in turn transferred into synthesize glutamate through the TCA cycle (Petroff, 2002). Recent publications indicate that the Glu-Gln-GABA cycle is involved in a variety of neurological disorders (Zheng et al., 2016, 2017; Lu et al., 2018; Zott et al., 2019). In the present study, the Glu levels showed no significant differences among three groups, however, the concentrations of Gln were brain region-specific, for example, compared with Con rats, the Gln level increased in all brain regions of PD rats and most significantly in the right cortex and right cerebellum, and it significantly increased in the midbrain and right cerebellum of LID rats but decreased in the right cortex. Our previous study showed that the disturbed Glu-Gln-GABA cycle occurred in the striatum of PD rats and bFGF treatment might alleviate PD symptoms through regulating these alterations (Zheng et al., 2016), another study reported that there was a significantly decreased striatal level of Glu in LID rats (Wang et al., 2018). Both higher or lower levels of Glu can be noxious, as excessive Glu can stimulate the activation of the NMDA receptor and induce excitatory toxicity (Jazvinšćak Jembrek et al., 2018).

Asp is another excitatory neurotransmitter in the central nervous system (Liu et al., 2013). Notably, the concentrations of Asp increased in all brain regions of rats in the experimental groups compared with the control group. Except for the right striatum and right hippocampus, the Asp level in PD group was significantly higher than that in the Con group and the Asp level in all brain regions had significant differences between Con and LID group. Since Glu, Gln and Asp are critical neurotransmitters, the brain-regional disturbance in neurotransmitters level observed in both PD and LID model could be considered a functional perturbation of Glu-Gln-GABA cycle in the synapse. We wondered if the PD as well as LID was associated with the changes of synaptic plasticity. In recent years, abnormal synaptic plasticity of cortical neurons was found to be causally involved in PD and LID (Ueno et al., 2017). Our results showed that the expression of SYP, a critical marker for synaptic structure and plastic plasticity (Zhu et al., 2019), significantly reduced in the right cortex in PD and LID rats. We, therefore, hypothesized that synaptic damage and neurotransmitter disturbance was involved in LID in parkinsonism, thus could be a potential target in the treatment or prevention of motor function impairment in PD and LID.

Meanwhile, osmoregulation plays an important role in maintaining cell structure and function. Myo is regarded as a marker for astrocytic and has various physiological functions such as membrane structure and osmoregulation (Isaacks et al., 1994; Edamatsu et al., 2018). In the present study, a significantly increased level of Myo in the midbrain and right hypothalamus of LID rats but no differences in PD rats as compared to normal rats may indicate the changed astrocytic activity underlying LID. Tau, another organic osmolyte in the brain, which was critical in osmoregulation and membrane stabilization (Trachtman et al., 1992). However, relative to Con rats, the concentration of Tau was only significantly decreased in the midbrain of PD rats but not in LID rats, suggesting that osmoregulation may be involved in PD. Thus, the disturbed osmoregulation as well as astrocytes-neurons crosstalk might involve in the occurrence of PD and LID.

The results of this study have detailed a widespread yet brain-regional specific metabolic disturbance in PD and LID rats. The midbrain and cortex exhibited significant metabolic disorders more than other brain regions. Moreover, various neurotransmitters and several intriguing metabolites, including glutamate, glutamine, aspartate, and myo-inositol were identified among the most discriminating metabolites. Our results suggest a possible mechanism: metabolic perturbation of neurotransmitter metabolism, especially in the synaptic Glu-Gln-GABA cycle may represent the pathological basis for the onset of PD and dyskinesia. While additional experiments will be necessary to firmly establish causal relations between metabolic disturbance and dyskinesia, these findings highlight the glutamate-glutamine cycle and synaptic plasticity in LID.

## Biosecurity Statement

All standard biosecurity and institutional safety procedures have been adhered to in all the experiment procedures in this article.

## Data Availability Statement

The datasets generated for this study are available on request to the corresponding author.

## Ethics Statement

The animal study was reviewed and approved by the Institutional Animal Care and Use Committee of Wenzhou Medical College (wydw2018-015).

## Author Contributions

HG, YY, and CY contributed to the experimental design. TZ, YX, QH, and CX contributed to animal experiments and behavior testing. TZ, WW, LZ, and HZ contributed to the sample collection, NMR metabolomic analysis, and synaptic plasticity analysis. CY, TZ, and HG contributed to the data analysis, result interpretation and writing. All authors have read, revised and approved the final manuscript.

## Supplementary Material

The Supplementary Material for this article can be found online at: https://www.frontiersin.org/articles/10.3389/fnagi.2020.00075/full#supplementary-material.

## Conflict of Interest

The authors declare that the research was conducted in the absence of any commercial or financial relationships that could be construed as a potential conflict of interest.

## Abbreviations

PD: Parkinson’s disease
Con: normal rats
LID: levodopa-induced dyskinesia
6-OHDA: 6-hydroxydopamine
AIMs: abnormal involuntary movements
Lac: lactate
Ala: alanine
GABA: γ-aminobutyric acid
NAA: N-acetyl aspartate
Gln: glutamine
Glu: glutamate
Asp: aspartate
Cre: creatine
Cho: choline
GPC: glycerol-phosphocholine
Tau: taurine
Myo: myo-inositol
Gly: glycine
ADP: adenosine diphosphate
AMP: adenosine monophosphate
Fum: fumarate
Mid: midbrain
Cor: cortex
Str: striatum
Hip: hippocampus
Cer: cerebellum
Hyp: hypothalamus.
