# Supplementary material for: Brain-Region Specific Metabolic Abnormalities in Parkinson’s Disease and Levodopa-Induced Dyskinesia
Source: Front Aging Neurosci. 2020 Mar 17;12:75. doi: 10.3389/fnagi.2020.00075 (PMC7089871; doi:10.3389/fnagi.2020.00075)
Supplement: Supplementary file 1 [file Table_1.docx]

**Supplemental Material**

Brain-region specific metabolic abnormalities in Parkinson’s disease and levodopa-induced dyskinesia

Changwei Yang^2#^, Tingting Zhang^1,2#^, Wuqiong Wang^2^, Yilan Xiang^1^, Qun Huang^1^, Chenglong Xie^3^, Liangcai Zhao^2^, Hong Zheng, Yunjun Yang^1^, Hongchang Gao^2^*

^1^School of Pharmaceutical Science, Wenzhou Medical University, Wenzhou 325035, China;

^2^Department of Radiology, The First Affiliated Hospital of Wenzhou Medical University, Wenzhou 325035, China;

^3^Department of Neurology, The First Affiliated Hospital of Wenzhou Medical University, Wenzhou 325035, China

^#^ Changwei Yang and Tingting Zhang contributed equally to this work.

**^*^Correspondence:**

Hongchang Gao

gaohc27@wmu.edu.cn (H.C. Gao);

Yunjun Yang (Y.J. Yang）

yyjunjim@163.com

**Table S1: Metabolite alterations in the midbrain of Con, PD and LID rats^1^**

| **Metabolite** | **Con^a^** | **PD^b^** | **LID^c^** |
| --- | --- | --- | --- |
| **Lactate** | 78.04±1.00 | 81.81±1.92 | 81.65±1.35 |
| **Alanine** | 4.95±0.10 | 4.63±0.10 | 4.63±0.09 ↓* |
| **NAA^d^** | 41.59±1.01 | 41.59±0.52 | 42.85±0.79 |
| **Glutamine** | 38.75±0.69 | 41.00±0.75 | 42.49±1.41 ↑* |
| **GABA^e^** | 22.03±0.45 | 21.09±0.45 | 19.94±0.75 |
| **Glutamate** | 29.65±0.73 | 29.79±0.74 | 32.11±0.90 |
| **Diamethylamine** | 3.63±0.21 | 3.29±0.14 | 3.51±0.50 |
| **Asparagine** | 6.29±0.11 | 6.78±0.15 ↑* | 6.87±0.13 ↑** |
| **Choline** | 14.39±0.36 | 13.91±0.42 | 12.86±0.71 |
| **Phosphocholine** | 8.01±0.15 | 7.83±0.12 | 7.76±0.18 |
| **GPC^f^** | 6.40±0.35 | 8.03±0.58 ↑* | 9.68±0.99 ↑* |
| **Taurine** | 11.08±0.30 | 10.15±0.23 ↓* | 11.04±0.0.37 |
| **Glycine** | 6.76±0.16 | 7.58±0.20 ↑* | 6.71±0.28 |
| **Myo-Inositol** | 30.35±0.42 | 31.63±0.44 | 32.69±0.55 ↑* |
| **Glu/Gln^g^**^,^**^h^** | 34.78±0.53 | 35.38±0.70 | 37.21±0.84 |
| **Creatine** | 37.07±0.53 | 37.37±0.30 | 37.97±0.63 |
| **ADP/AMP^i^** | 2.79±0.08 | 2.59±0.18 | 2.23±0.25 |
| **Fumarate** | 0.30±0.01 | 0.31±0.02 | 0.31±0.01 |
| **AMP^j^** | 0.69±0.03 | 0.88±0.10 | 1.25±0.22 ↑* |
| **Inosine** | 3.00±0.07 | 2.83±0.19 | 2.60±0.21 |

^1^“↑” or “↓” means the concentration of the metabolite in the PD or LID group increased or decreased compared with Con group. ^∗^*P* < 0.05, ^∗∗^*P* < 0.01.

^a^ normal control rats; ^b^ 6-OHDA-lesioned rats; ^c^ L-dopa induced dyskinesia rats; ^d^

N-acetylaspartate; ^e^ γ-Aminobutyric acid; ^f^ glycerol-phosphocholine; ^g^ Glu: glutamate; ^h^ Gln: glutamine; ^i^ ADP: adenosine diphosphate; ^j^ adenosine monophosphate**.**

**Table S2: Metabolite alterations in the right striatum of Con, PD and LID rats^1^**

| **Metabolite** | **Con ^a^** | **PD ^b^** | **LID ^c^** |
| --- | --- | --- | --- |
| **Lactate** | 67.13±2.50 | 75.22±1.07 ↑* | 73.10±0.73 ↑* |
| **Alanine** | 7.16±0.22 | 6.86±0.21 | 7.28±0.26 |
| **NAA ^d^** | 35.49±1.37 | 36.67±1.01 | 37.66±1.07 |
| **Glutamine** | 45.86±1.87 | 48.86±0.63 | 47.78±1.20 |
| **GABA ^e^** | 20.21±1.98 | 19.29±0.48 | 21.73±1.47 |
| **Glutamate** | 33.58±2.20 | 36.23±0.87 | 35.14±1.37 |
| **Diamethylamine** | 7.67±0.51 | 8.69±0.49 | 8.00±0.51 |
| **Asparagine** | 4.97±0.15 | 5.15±0.10 | 5.70±0.11 ↑** |
| **Choline** | 15.33±1.58 | 13.29±1.08 | 12.52±1.30 |
| **Phosphocholine** | 17.79±2.26 | 13.89±0.99 | 11.88±1.31 ↓* |
| **GPC ^f^** | 6.35±0.26 | 6.69±0.47 | 6.85±0.63 |
| **Taurine** | 30.99±1.02 | 30.34±0.96 | 29.16±0.77 |
| **Glycine** | 5.70±0.48 | 5.54±0.39 | 4.81±0.36 |
| **Myo-Inositol** | 25.36±0.36 | 25.7±0.41 | 25.72±0.49 |
| **Glu/Gln ^g,h^** | 40.03±1.58 | 41.21±0.86 | 41.60±1.23 |
| **Creatine** | 36.27±0.72 | 37.22±0.67 | 37.90±0.83 |
| **ADP/AMP ^i^** | 2.73±0.17 | 2.44±0.25 | 2.11±0.26 |
| **Fumarate** | 0.35±0.01 | 0.36±0.02 | 0.35±0.01 |
| **AMP ^j^** | 1.27±0.21 | 1.64±0.20 | 1.90±0.26 |
| **Inosine** | 3.88±0.19 | 3.57±0.33 | 3.38±0.23 |

^1^“↑” or “↓” means the concentration of the metabolite in the PD or LID group increased or decreased compared with Con group. ^∗^*P* < 0.05, ^∗∗^*P* < 0.01.

^a^ normal control rats; ^b^ 6-OHDA-lesioned rats; ^c^ L-dopa induced dyskinesia rats; ^d^

N-acetylaspartate; ^e^ γ-Aminobutyric acid; ^f^ glycerol-phosphocholine; ^g^ Glu: glutamate; ^h^ Gln: glutamine; ^i^ ADP: adenosine diphosphate; ^j^ adenosine monophosphate.

**Table S3: Metabolite alterations in the right cortex of Con, PD and LID rats^1^**

| **Metabolite** | **Con ^a^** | **PD ^b^** | **LID ^c^** |
| --- | --- | --- | --- |
| **Lactate** | 79.07±2.50 | 82.61±1.69 | 82.99±2.57 |
| **Alanine** | 6.00±0.06 | 5.71±0.14 | 5.99±0.18 |
| **NAA ^d^** | 51.09±1.49 | 51.23±1.08 | 49.98±1.22 |
| **Glutamine** | 52.59±1.07 | 56.25±0.60 ↑* | 52.46±1.00 |
| **GABA ^e^** | 13.14±0.32 | 11.73±0.19 ↓** | 13.45±1.39 |
| **Glutamate** | 46.52±1.31 | 48.15±0.91 | 44.53±1.20 |
| **Diamethylamine** | 1.86±0.12 | 1.69±0.11 | 1.57±0.09 |
| **Asparagine** | 6.29±0.13 | 6.69±0.08 ↑* | 6.85±0.12 ↑* |
| **Choline** | 8.30±0.25 | 7.59±0.31 | 8.09±0.47 |
| **Phosphocholine** | 6.84±0.39 | 6.47±0.18 | 6.64±0.44 |
| **GPC ^f^** | 6.07±0.25 | 7.8±0.30 ↑** | 7.83±0.57 ↑* |
| **Taurine** | 22.34±0.60 | 22.07±0.89 | 22.15±0.54 |
| **Glycine** | 3.91±0.14 | 3.82±0.08 | 3.89±0.17 |
| **Myo-Inositol** | 26.54±1.07 | 25.79±0.86 | 27.68±0.93 |
| **Glu/Gln ^g,h^** | 47.45±1.01 | 49.52±0.54 | 47.12±0.66 |
| **Creatine** | 38.47±0.59 | 37.85±0.56 | 37.11±0.92 |
| **ADP/AMP ^i^** | 1.10±0.10 | 0.98±0.10 | 1.02±0.19 |
| **Fumarate** | 0.32±0.01 | 0.24±0.01 ↓*** | 0.31±0.02 |
| **AMP ^j^** | 2.89±0.14 | 2.97±0.11 | 2.87±0.17 |
| **Inosine** | 2.08±0.06 | 1.87±0.13 | 1.92±0.23 |

^1^“↑” or “↓” means the concentration of the metabolite in the PD or LID group increased or decreased compared with Con group. ^∗^*P* < 0.05, ^∗∗^*P* < 0.01, ^∗*∗^*P* < 0.001.

^a^ normal control rats; ^b^ 6-OHDA-lesioned rats; ^c^ L-dopa induced dyskinesia rats; ^d^

N-acetylaspartate; ^e^ γ-Aminobutyric acid; ^f^ glycerol-phosphocholine; ^g^ Glu: glutamate; ^h^ Gln: glutamine; ^i^ ADP: adenosine diphosphate; ^j^ adenosine monophosphate.

**Table S4: Metabolite alterations in the right hippocampus of Con, PD and LID rats^1^**

| **Metabolite** | **Con ^a^** | **PD ^b^** | **LID ^c^** |
| --- | --- | --- | --- |
| **Lactate** | 86.75±1.24 | 86.65±1.59 | 83.93±1.53 |
| **Alanine** | 6.87±0.16 | 6.89±0.23 | 6.70±0.08 |
| **NAA ^d^** | 42.56±0.7 | 42.11±0.81 | 43.31±0.49 |
| **Glutamine** | 46.92±1.07 | 49.58±1.18 | 48.78±1.00 |
| **GABA ^e^** | 16.01±0.74 | 15.51±0.99 | 14.23±0.34 ↓* |
| **Glutamate** | 39.42±1.22 | 40.55±1.43 | 41.23±0.72 |
| **Diamethylamine** | 0.77±0.01 | 0.74±0.02 | 0.73±0.01 |
| **Asparagine** | 4.78±0.05 | 4.95±0.10 | 5.10±0.04 ↑** |
| **Choline** | 7.99±0.62 | 8.02±0.97 | 7.51±0.41 |
| **Phosphocholine** | 8.54±0.22 | 8.63±0.48 | 8.10±0.23 |
| **GPC ^f^** | 7.19±0.33 | 7.72±0.64 | 8.23±0.23 ↑* |
| **Taurine** | 22.63±0.78 | 21.78±0.77 | 22.29±0.46 |
| **Glycine** | 4.68±0.20 | 5.04±0.29 | 4.49±0.09 |
| **Myo-Inositol** | 32.12±0.62 | 32.22±0.47 | 32.95±0.46 |
| **Glu/Gln ^g,h^** | 44.11±0.83 | 45.76±0.89 ↑* | 46.13±0.54 |
| **Creatine** | 37.44±0.46 | 38.26±0.33 | 38.09±0.43 |
| **ADP/AMP ^i^** | 1.27±0.20 | 1.31±0.19 | 1.13±0.13 |
| **Fumarate** | 0.33±0.02 | 0.33±0.02 | 0.28±0.01 ↓* |
| **AMP ^j^** | 2.21±0.24 | 2.18±0.17 | 2.31±0.10 |
| **Inosine** | 2.61±0.14 | 2.51±0.31 | 2.26±0.22 |

^1^“↑” or “↓” means the concentration of the metabolite in the PD or LID group increased or decreased compared with Con group. ^∗^*P* < 0.05, ^∗∗^*P* < 0.01.

^a^ normal control rats; ^b^ 6-OHDA-lesioned rats; ^c^ L-dopa induced dyskinesia rats; ^d^

N-acetylaspartate; ^e^ γ-Aminobutyric acid; ^f^ glycerol-phosphocholine; ^g^ Glu: glutamate; ^h^ Gln: glutamine; ^i^ ADP: adenosine diphosphate; ^j^ adenosine monophosphate.

**Table S5: Metabolite alterations in the right cerebellum of Con, PD and LID rats^1^**

| **Metabolite** | **Con ^a^** | **PD ^b^** | **LID ^c^** |
| --- | --- | --- | --- |
| **Lactate** | 91.81±2.23 | 93.49±1.55 | 91.30±1.89 |
| **Alanine** | 5.42±0.15 | 5.34±0.05 | 5.16±0.06 |
| **NAA ^d^** | 39.67±0.53 | 39.76±0.58 | 40.48±0.49 |
| **Glutamine** | 48.36±1.06 | 52.60±0.58 ↑** | 51.16±0.64 ↑* |
| **GABA ^e^** | 11.91±0.62 | 11.04±0.35 | 10.33±0.37 ↓* |
| **Glutamate** | 37.83±1.08 | 39.02±0.49 | 38.96±0.44 |
| **Diamethylamine** | 1.44±0.08 | 1.67±0.07 | 1.69±0.09 |
| **Asparagine** | 4.95±0.08 | 5.30±0.04 ↑** | 5.49±0.10 ↑** |
| **Choline** | 8.53±0.56 | 8.36±0.50 | 7.35±0.42 |
| **Phosphocholine** | 6.87±0.09 | 6.62±0.22 | 6.56±0.18 |
| **GPC ^f^** | 7.07±0.96 | 7.59±0.86 | 8.95±0.53 |
| **Taurine** | 18.88±0.42 | 18.24±0.39 | 18.05±0.38 |
| **Glycine** | 4.50±0.50 | 4.65±0.25 | 4.42±0.19 |
| **Myo-Inositol** | 31.38±0.41 | 30.50±0.24 | 31.82±0.49 |
| **Glu/Gln ^g,h^** | 44.10±0.86 | 45.35±0.38 | 45.26±0.32 |
| **Creatine** | 50.08±0.61 | 51.18±0.77 | 50.89±0.53 |
| **ADP/AMP ^i^** | 1.51±0.24 | 1.47±0.20 | 1.11±0.17 |
| **Fumarate** | 0.25±0.01 | 0.26±0.01 | 0.26±0.01 |
| **AMP ^j^** | 2.08±0.31 | 2.22±0.17 | 2.55±0.16 |
| **Inosine** | 2.70±0.23 | 2.68±0.33 | 2.29±0.27 |

^1^“↑” or “↓” means the concentration of the metabolite in the PD or LID group increased or decreased compared with Con group. ^∗^*P* < 0.05, ^∗∗^*P* < 0.01.

^a^ normal control rats; ^b^ 6-OHDA-lesioned rats; ^c^ L-dopa induced dyskinesia rats; ^d^

N-acetylaspartate; ^e^ γ-Aminobutyric acid; ^f^ glycerol-phosphocholine; ^g^ Glu: glutamate; ^h^ Gln: glutamine; ^i^ ADP: adenosine diphosphate; ^j^ adenosine monophosphate.

**Table S6: Metabolite alterations in the hypothalamus of Con, PD and LID rats^1^**

| **Metabolite** | **Con ^a^** | **PD ^b^** | **LID ^c^** |
| --- | --- | --- | --- |
| **Lactate** | 81.02±2.66 | 79.62±6.7 | 83.71±1.76 |
| **Alanine** | 5.38±0.23 | 5.50±0.24 | 5.06±0.22 |
| **NAA ^d^** | 33.01±1.48 | 30.49±2.82 | 34.71±0.83 |
| **Glutamine** | 39.08±1.09 | 38.33±2.86 | 42.58±1.59 |
| **GABA ^e^** | 26.01±0.83 | 25.91±2.6 | 25.71±1.46 |
| **Glutamate** | 23.03±0.89 | 20.87±1.96 | 25.37±1.39 |
| **Diamethylamine** | 2.45±0.20 | 3.01±0.48 | 2.54±0.11 |
| **Asparagine** | 5.37±0.12 | 6.10±0.13 ↑** | 6.09±0.07 ↑*** |
| **Choline** | 13.87±0.56 | 16.15±1.83 | 15.78±2.02 |
| **Phosphocholine** | 11.10±0.30 | 10.76±1.12 | 11.06±0.40 |
| **GPC ^f^** | 13.17±0.68 | 11.81±2.03 | 16.39±1.66 |
| **Taurine** | 9.47±0.17 | 10.42±1.23 | 9.96±0.37 |
| **Glycine** | 7.21±0.81 | 7.18±0.52 | 6.18±0.30 |
| **Myo-Inositol** | 34.41±0.88 | 32.15±2.68 | 37.41±0.68 ↑* |
| **Glu/Gln ^g,h^** | 33.15±1.00 | 31.30±2.71 | 36.13±1.11 |
| **Creatine** | 27.91±1.00 | 25.97±2.36 | 29.18±0.39 |
| **ADP/AMP ^i^** | 2.29±0.10 | 2.21±0.13 | 1.95±0.30 |
| **Fumarate** | 0.26±0.01 | 0.34±0.06 | 0.28±0.02 |
| **AMP ^j^** | 0.64±0.07 | 0.86±0.18 | 1.33±0.39 |
| **Inosine** | 2.39±0.08 | 2.24±0.13 | 2.08±0.26 |

^1^“↑” or “↓” means the concentration of the metabolite in the PD or LID group increased or decreased compared with Con group. ^∗^*P* < 0.05, ^∗∗^*P* < 0.01, ^∗*∗^*P* < 0.001.

^a^ normal control rats; ^b^ 6-OHDA-lesioned rats; ^c^ L-dopa induced dyskinesia rats; ^d^

N-acetylaspartate; ^e^ γ-Aminobutyric acid; ^f^ glycerol-phosphocholine; ^g^ Glu: glutamate; ^h^ Gln: glutamine; ^i^ ADP: adenosine diphosphate; ^j^ adenosine monophosphate.
